# Recent Advances on Pickering Emulsions Stabilized by Diverse Edible Particles: Stability Mechanism and Applications

**DOI:** 10.3389/fnut.2022.864943

**Published:** 2022-05-06

**Authors:** Wei Li, Bo Jiao, Sisheng Li, Shah Faisal, Aimin Shi, Weiming Fu, Yiying Chen, Qiang Wang

**Affiliations:** ^1^Institute of Food Science and Technology, Chinese Academy of Agriculture Sciences/Key Laboratory of Agro-Products Processing, Ministry of Agriculture and Rural Affairs, Beijing, China; ^2^College of Food Science and Engineering, Nanjing University of Finance and Economics/Collaborative Innovation Center for Modern Grain Circulation and Safety, Nanjing, China

**Keywords:** Pickering emulsion, food-grade particles, stabilization mechanism, particles shapes, food application

## Abstract

Pickering emulsions, which are stabilized by particles, have gained considerable attention recently because of their extreme stability and functionality. A food-grade particle is preferred by the food or pharmaceutical industries because of their noteworthy natural benefits (renewable resources, ease of preparation, excellent biocompatibility, and unique interfacial properties). Different edible particles are reported by recent publications with distinct shapes resulting from the inherent properties of raw materials and fabrication methods. Furthermore, they possess distinct interfacial properties and functionalities. Therefore, this review provides a comprehensive overview of the recent advances in the stabilization of Pickering emulsions using diverse food-grade particles, as well as their possible applications in the food industry.

## Introduction

Pickering emulsions are stabilized by solid or soft nano (micro) particles. They are first discovered by Ramsden (1) in 1903 that the mixture of wax and water could be stabilized by solids, thus forming emulsions. Subsequently, Pickering (2) put forward to stabilize emulsions by using nanoparticles and microparticles, which promoted the progress of emulsion research. Hence, the emulsion stabilized by particles was identified and described as “Pickering emulsion”, which can be oil-in-water (O/W), water-in-oil (W/O), water-in-water (W/W) (3), or even multiple (4–6). There are prime differences in the behavior of the low-molecular-weight surfactants and natural polymers, particles with sizes ranging from just several nanometers to micrometers during the preparation and stabilization of the Pickering emulsions. Particles can provide a space barrier between the two immiscible phases to prevent droplets' coalescence (7) and Ostwald ripening in Pickering emulsions (8). Some researchers have provided different stability functions of particles in Pickering emulsion. Tavasoli et al. (9) considered that the capillary pressure due to the anisotropic shapes of solid particles can prevent the interface film drainage. Dickinson et al. (9, 10) presented a fact that the form of a network of particles in the continuous phase can generate a barrier to improving the stability of many gel-like emulsions of moderately high oil volume fraction. These all could provide significantly higher stability.

Initially, Pickering emulsions did not draw enough attention because of the limitation of materials which showed partial wetting in both phases. Subsequently, Pickering emulsion stabilized by inorganic solid nanoparticles such as silica particles (11), polymer lattices (12), and clay (13) received a wide scope of research attention because of the development of chemical engineering and material science (14). Furthermore, it has been applied to some novel areas, such as oil recovery. However, their applications in cosmetic products, food technology, as well as pharmaceutical industries were extremely limited due to their biodegradability and biocompatibility concerns. Recently, with the development of biomacromolecules materials science, varieties of novel particles with adjustable surface wettability and better biocompatibility were used to stabilize Pickering emulsions (15). Particles made of natural edible resources such as polysaccharides (16, 17), protein (18) have been broadly used to stabilize Pickering emulsions to satisfy diverse needs, from food and cosmetic industries to catalysis, tissue engineering, and drug delivery.

The shapes of the particles can be different due to the diverse nature, composition, and structure of these biopolymers. Specifically, the behavior at the interface and the ability to stabilize the emulsions can be governed by the shape of particles (19). Some researchers (20, 21) considered that due to their higher aspect ratio of anisotropic particles, the interfacial layer, the desorption energy value, and the capillary force can all be increased leading to more stable emulsion systems. The research and development of particles with different shapes have become a hotspot. The novel shape of solid particles, including ellipsoids, nanofibrils, nanocages, plated-shape, nanotubes, and other irregular shapes (22), could exhibit different stabilization mechanisms of Pickering emulsion. We explicitly dedicated a section summarizing and discussing other mechanisms for stabilization of Pickering emulsions other than the irreversible adsorption behavior of particles at the oil-water interface and the physical obstacle especially the stability mechanism of high internal phase Pickering emulsions (HIPEs). It is also worth noting that, Pickering emulsion catalysis has advanced rapidly in the past decades, some potential applications have been found in the food area. However, no review has been published in the food area contained the application aspect of Pickering emulsion catalysis. To comprehensively review the recent advances on Pickering emulsions stabilized by diverse edible particles, besides the novel points we mentioned above, an integrated overview of the stabilization mechanism and application of Pickering emulsions have been given. Furthermore, an outlook on this topic has been raised as well. We believe this review could provide the latest progress and new insight into the field of Pickering emulsions readers.

## The Stabilization Mechanism of Pickering Emulsions

### The Irreversible Adsorption and Robust Physical Barriers of Particles at the Oil-Water Interface

**Figure 1:**
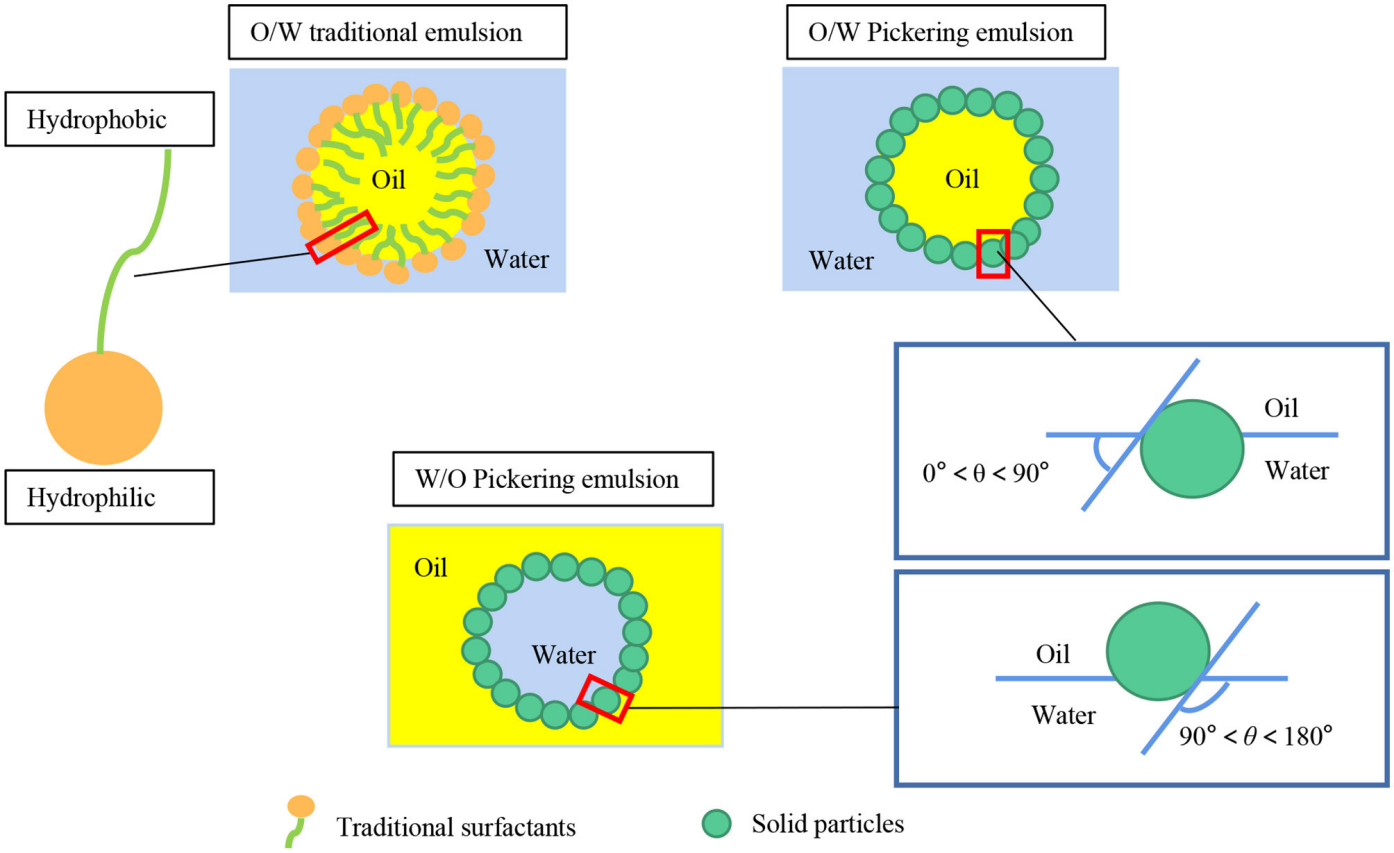
Sketch of traditional emulsions and Pickering emulsions.

The mechanism of Pickering emulsions is different from emulsions stabilized by traditional surfactants (Figure 1) and biopolymers with hydrophilic and hydrophobic two distinct regions. The most common one is that Pickering emulsions are stabilized through robust interface adsorption by particles so it does not need to be amphiphilic (23). The wettability of particles is critical for particles to adsorb at the surface of the droplets and the final stabilization of Pickering emulsions. To understand the difference between them, it is necessary to explain the irreversible adsorption and robust physical barriers of particles at the oil-water interface.

The strong adsorption is driven by the partial wettability of certain spherical hard solid particles, lead to firm steric hindrance. Therefore, it can prevent the emulsion droplets from flocculation and coalescence by a steric mechanism. Adsorption of particles at the oil-water interface requires partial wetting by both the oil and water phases (24). And the wettability can be measured by contact angles (θ). According to the contact angles (θ) of solid particles, the emulsions are classified into different types. O/W emulsions (θ < 90°) can be formed by particles easily wetted by water, otherwise, W/O emulsions (90° < θ <180°) are fabricated.

Desorption of particles from the oil-water interface requires overcoming high interfacial energy, so a firm space barrier to prevent droplet coalescence can be formed at the oil-water interface. Thus, Pickering emulsions are endowed with much better stability than traditional emulsions stabilized by surfactants. This is a matter of interfacial energies of the three interfaces: solid-oil, solid-water, and water-oil abbreviated γ_s−o_, γ_s−w_, γ_w−o_. O/W emulsions require positive adhesion energy of oil E_Adh_(O/W), and negative spreading coefficient of oil S(O/W) (23), (Equations 1, 2):


(1) $$E_{Adh}(O/W)=\gamma_{s-w}+\gamma_{w-o}-\gamma_{s-o}>0$$

(2) $$s(o/w)=\gamma_{s-w}-\gamma_{w-o}-\gamma_{s-o}<0$$

Formation of the W/O emulsion needs the adsorption energy of water, E_Adh_ (W/O) keeping positive, and S (W/O) remaining negative. When the surface of solid particles is entirely hydrophilic, it will be completely wetted by the water phase. Solid particles will not adsorb at the water-oil interface. They are completely dispersed in the water phase, where θ = 0°. Similarly, the solid particles are completely wetted by oil, where θ =180°. Therefore, emulsions tend to be formed only when particles are partially wetted. Besides Pickering emulsions will be more stable if the contact angle is preferentially close to 90°. One most common practiced methods of the contact angle is the conventional Young's equation (θ_o_=90°-θ_w_) (25). The contact angle in the water phase is θ_w_, and the contact angle in the oil phase is θ_o_, (Equations 3, 4):


(3) $$cos(\theta_{w})=\left(\gamma_{s-o}-\gamma_{s-w}\right)/\gamma_{w-o}$$

(4) $$cos(\theta_{o})=(\gamma_{s-w}-\gamma_{s-o})/\gamma_{w-o}$$

Since the solid particles for the stabilization of Pickering particles are small, their gravity and buoyancy can be negligible. The detachment energy E required for desorption from the interface of a spherical solid particle can be calculated using the formula (5) below (26):


(5) $$E=\pi r^{2}\gamma_{w-o}{\left(1-\left|\cos\theta \right|\right)}^{2}$$

Where r is the radius of solid spherical particles stabilizing the emulsion, γ_W−O_ is the surface tension of oil-water interface, and θ is the three-phase contact angles. For instance, in a solid particle with a radius of 10 nm, when the contact angle is 90°, the spontaneous detachment energy required for desorption from the interface enormously exceeds the thermal energy of Brownian motion (27), which also explains why the adsorption of solid particles at the interface is irreversible. Based on the formula, when the contact angle is too close to 0° or 180°, the thermal energy is greater than the detachment energy. As a result, the solid particles cannot permanently adsorb at the interface to stabilize the Pickering emulsion effectively.

### The Capillary Pressure Between Particles

**Figure 2:**
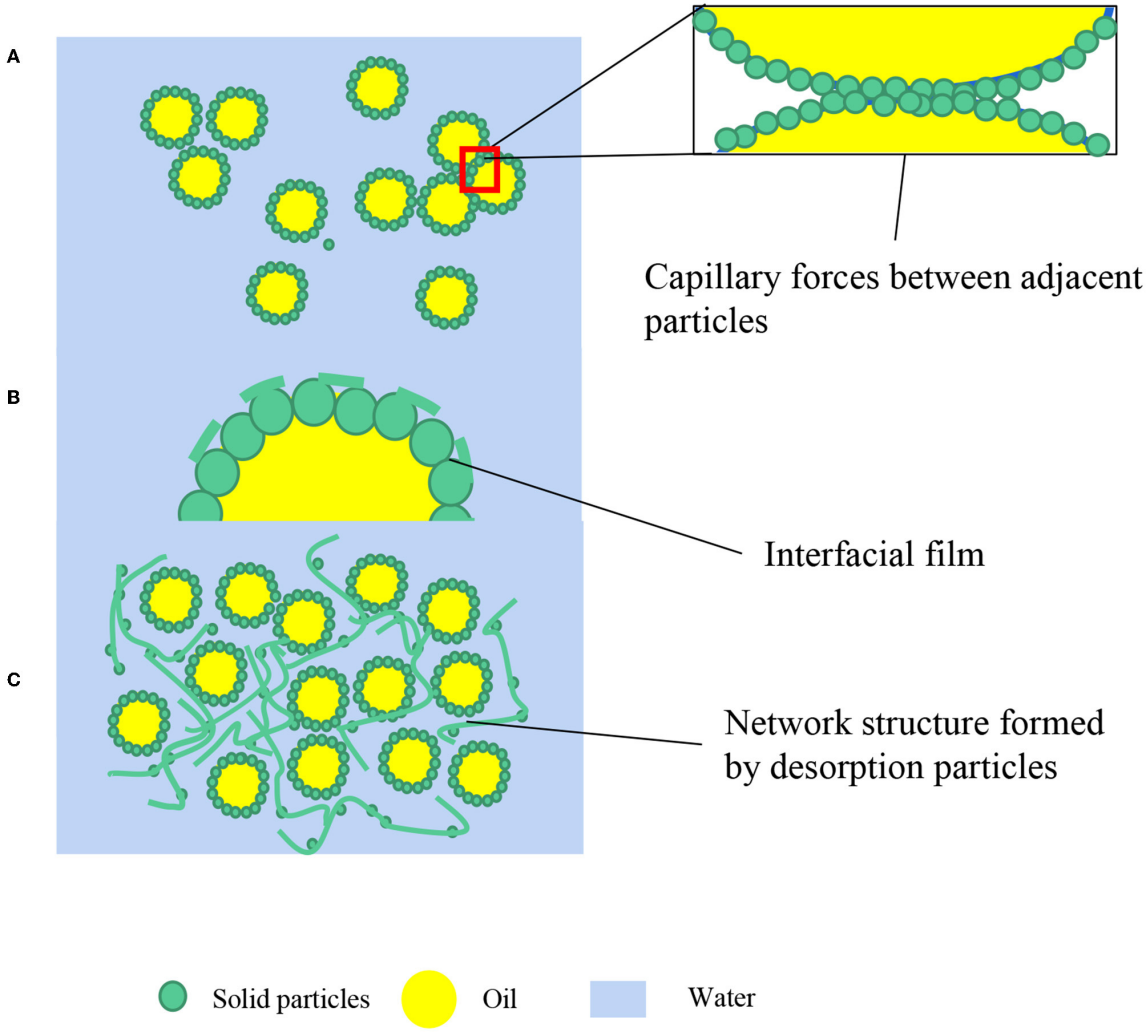
Different stabilization mechanisms of Pickering emulsions. **(A)** Capillary forces between adjacent particles; **(B)** Dense interfacial film; **(C)** Network structure in a continuous phase.

The capillary pressure (Figure 2A) between adjacent particles at the interface can prevent thin film formed by particles degradation. The non-spherical particles (rod-like, disk-like, fibers, etc.) may fabricate more stable emulsion systems. One of the reasons has been revealed that anisotropic particles with different geometrical shapes can generate stronger capillary pressure between adjacent particles at the interface (20, 21). The details have been elaborated in Section Pickering Emulsions Stabilized by Different Shapes of Food-Grade Particles.

### The Interfacial Rheological Responses

The interfacial rheological responses are related to the interfacial adsorption and the interaction amongst particles (28) and can affect film (Figure 2B) drainage. The interfacial structure may be transformed into the multilayer arrangement, with one or more extra layers of ordered monodisperse hard spheres accommodated in the interdroplet region, where a substantial excess of particles exists in the continuous phase (29). The additional particle layering can lead to an oscillatory potential of mean force and an associated kinetic structural barrier that can protect droplets from coalescence (30). This layering formed by particles that are not adsorbed at the oil-water interface is common, especially in a host of colloidal particles, such as self-assemble entities that are neither fully monodispersed nor closely resembling hard spherical particles.

### The Formation of Particles' Network in the Space Between Droplets

Another important stabilization principle is the intrinsic ability of microgels to act as a versatile thickener agent and colloid stabilizer in an aqueous medium (10). The more stable and highly viscous structure can stabilized HIPEs stabilized by biopolymers due to a self-supporting gel network (Figure 2C) (31). In this way, these HIPEs are able to have resistance to good freeze-thaw reversibility and drastic heating. The particles are predominantly playing the role of a “structure agent” rather than necessarily adsorbing at the oil-water interface (32). Xu et al. (33) used bovine serum albumin glycated with galactose possessing much structural integrity and higher refolding ability that can improve the stability of HIPEs compared to native bovine serum albumin. Additionally, Rayner et al. (34) further found that some adsorbed layers of biopolymer particles at the oil-water interface can be made to fuse together into a coherent gel-like lipid-encapsulating layer through post-emulsification thermal processing.

## Pickering Emulsions Stabilized by Different Shapes of Food-Grade Particles

In recent years, food-grade particles of different shapes have attracted researchers' attention. Zhang et al. (22) summarized protein nanoparticles with different shapes, and various preparation processes of them are also reviewed. However, many natural polymers other than proteins can be used to prepare particles with various shapes through different modification methods. Common modification ways include octenyl succinic anhydride (OSA) modification of starch, protein modification (enzymatic modification, phosphorylation modification, aldehyde modification, and phenolic modification) (35, 36), and preparation of electrostatic complexes. The particle shapes with different shapes which have various particle densities, desorption energy, and capillary forces between adjacent particles largely affect the stabilization with different principles and application of emulsions (Table 1). Particle shapes can be divided into regular spherical and irregular shapes with anisotropic morphology (25). The causes of different solid particle shapes are related to their sources, properties, structures, and particle preparation methods (22). In addition, particle shape can critically impact the stabilization of emulsion by modifying the wettability behavior of particles and the interactions between adjacent particles.

**Table 1:** Different shapes of food-grade particles.

**Morphology**	**Materials**	**Particles size**	**Emulsion type**	**Application**	**References**
Spherality	Green tea polyphenol	100–400 nm diameter	O/W	Enhance the oxidability of emulsion	(37)
	Rice peptide	357.8 nm diameter	O/W	Improve oxidative stability of emulsion	(38)
	Zein-chitosan	—	O/W	Encapsulation of VD_3_	(39)
	Hordein-chitosan	594.9 nm diameter	O/W	Encapsulation of active substances and replacing existing food system	(40)
	Zein-pullulan complex	147.4 nm diameter without pullulan; 594.9 nm diameter with pullulan	O/W	Encapsulation and releasing of active substances	(41)
	Luteolin	125.6 ± 24.7 nm diameter	O/W	Enhance the oxidability of pine nut oil	(42)
	Amylopectin-xyloglucan	184 ± 7 nm diameter	O/W	—	(43)
	Zein	About 200 nm diameter	—	—	(44)
	nanocellulose	≤ 150 nm diameter	O/W	Encapsulation of curcumin and coumarin	(45)
Rod-like	Cellulose nanocrystals	several microns length 30.61 ± 6.67 nm width	O/W	Improving the storage stability of emulsion against high temperature and sheering	(46)
	Cellulose nanocrystals	The needle-like morphology with about 200 nm length and 10 nm diameter CNCs modified by BSA with 500 nm length and 30 nm width	O/W	Broden the application of food and drugs in the central nervous system	(47)
	Cellulose colloidal nanorods	185 nm length; 13–160 aspect radio		—	(48)
Ellipsoid	Cellulose nanocrystals	—	O/W	—	(49)
	Polystyrene	Long axis 11–45 μm Short axis 4.5–9 μm	O/W	—	(50)
Long ellipsoid	Polystyree	Aspect radio 1:9	O/W	—	(51)
Spindle		Aspect radio 1:6	W/O		
Nanofibrils	Nanocellulose	20 nm average diameter, 2.0 ± 0.5 nm length, aspect radio 100	O/W	Food storage, food emulsifier, active food packing, wound healing, surface biocides and antibacterial cleaning products	(52)
	Nanocellulose	200 nm length, 8 nm width	O/W	Application in food and drugs	(53)
	Nanocrystals	—	O/W	Replacing of existing food system	(52)
Nanocages	E2 protein	25 nm diameter with 12 openings of about 5 nm	O/W	Encapsulation of new food and cosmetics	(54)
Plate-Shaped	wool keratin	—	O/W and W/O	Carrying new drugs and cosmetics	(55, 56)
Nanotubes	α-lac-protein	20–30 nm diameter; 0.5–1 μm length	O/W	Releasing flavor fatty acid	(57)
Surface depression	Spray drying soy protein	20–60 μm	O/W	—	(58)
Spongy	Plant spore	20–60 μm	O/W	—	(59)
Flaky	Phytosterol	30–35 μm	O/W	—	(60)

### Spherical Solid Particles

Sphere is one of the most common shapes of food-grade particles, which has been widely used in drug delivery and food products due to its easy preparation and low cost (61). Both undeformable solid spherical particles and porous microspheres belong to common food-grade spherical solid particles, whose diameters are shorter than 400 nm. Among them, the mechanism of undeformable spherical particles stabilizing Pickering emulsions has been described in Section Introduction. Spherical particles are mostly formed by protein, polysaccharide, and other macromolecules crimped by intramolecular forces. The spherical particles formed by a single natural molecule are not regular enough. Hence more extra substances are needed to form complexes to improve the better anti-aggregation properties of solid particles. Shah (39) and Li (40) modified zein and barley gliadin with chitosan to formulate spherical solid particles with obvious anti-aggregation and anti-destabilization ability for embedding active substances respectively. Liu et al. (62) used epigallocatechin gallate (EGCG) connected with zein nanoparticles to form covalent or non-covalent through alkali treatment. Experimental results showed the protein-polyphenol complexes had a smooth surface and reduced emulsion droplet size causing better emulsion stability. Polyphenols alter the hydrophobic and electrostatic interactions among protein molecules, giving higher thermal stability and antioxidant capacity of the complex. Soybean protein is often used to prepare regular spherical solid particles to meet various needs through some processes such as pH shift (63, 64), heating treatment (65, 66), enzymatic hydrolysis (67, 68), denatured agent induction [urea (61, 69), ethanol (70), treatment, etc.], and hydrostatic high-pressure treatment (71). These denatured processes can lead to alteration in protein structure, thus, triggering self-assembly or mutual assembly behavior.

### Rod-Like Solid Particles

Regular rod, prolate ellipsoid, and spindle rod are common rod-like shapes of solid particles, often prepared by using cellulose nanocrystals (CNC) with the material of rod-shaped particles, cellulosic colloidal nano-rods (48), etc. These particles have relatively sufficient aspect ratios able to stabilize Pickering emulsions effectively (51). Cellulose is a linear homopolysaccharide composed of repeating units of β-1, 4-D-glucopyranose, and nanocellulose, which refers to nanoscale cellulose with a diameter ranging from 1 to 100 nm (72), which can be obtained from agricultural wastes, such as cotton and wood fibers. Common types of nanocellulose include micro-fibrillated cellulose (MFC), bacterial nanocellulose (BNC), and nanocrystalline cellulose (NCC) (73). The specific surface area, high strength, low density, and amphiphilic ability of nanocellulose provide conditions for stabilizing Pickering emulsions (52). Solid particles with different shapes can be prepared by using nanocellulose from different sources, and they are mainly rod-shaped (74).

**Figure 3:**
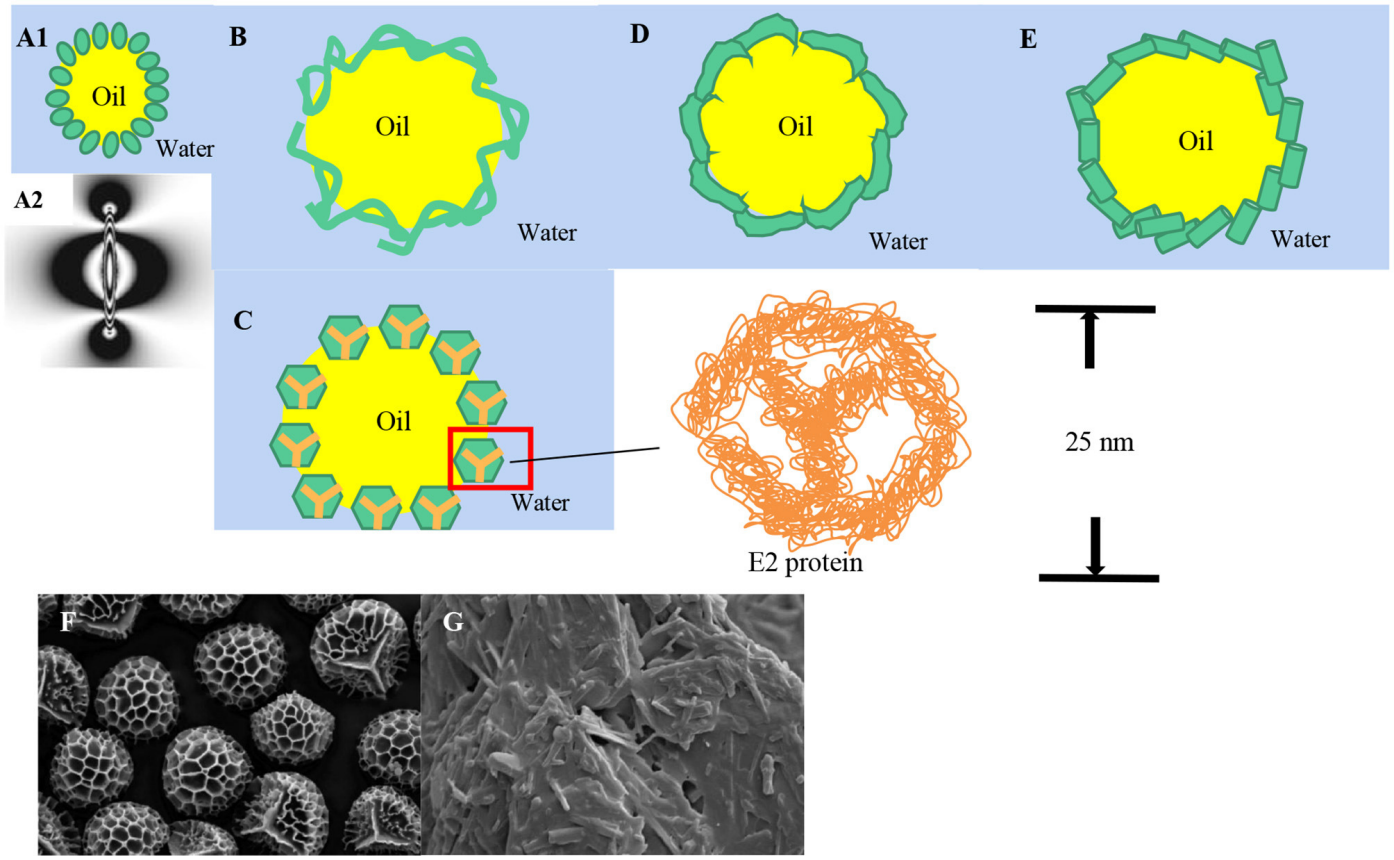
Food-grade particles are of different shapes. **(A)** Interface deformation of ellipsoidal particles; **(B)** Nanofibrils particles; **(C)** E2 protein nanocages; **(D)** Disk solid particles; **(E)** Nanotubes; **(F)** SEM image of dry Lycopodium clavatum spore; **(G)** SEM micrographs of original phytosterol. (Reused from Liu et al. [(50, 59, 60) with permission].

It was found that the principle of stable Pickering emulsion of rod-shaped particles was different from that of regular spherical particles. The adsorption of prolate ellipsoids on the water-oil interface mainly depends on two forces. One is the capillary interactions brought about the interface deformation from the overlap between particles (75). The other is the fluctuation-induced force caused by thermally excited capillary waves arising at fluid interfaces (49). Gurappa et al. (51) found that the interface with controlled surface rheology could be obtained by using shape-induced capillary force as well as packing effects. The capillary force affects the aggregation of bubbles and solid particles at the gas-water interface (76). For slightly heavier particles, the capillarity force comes from interface deformation caused by gravity (75). For exceptionally light particles, although gravity is ignored, capillary action still exists at the interface for electrostatic interaction among particles (77, 78). It may also be because the particles are irregularly spherical solid particles, resulting in different degrees of wetting (79–81). The capillary force generated by the deformation of the interface caused by the small particles can be both mutually attractive and mutually repellant, and the interface deformation gets much larger. Thus, the interaction becomes much stronger (82). Loudet et al. (75) investigated the principle and interface characteristics of Pickering emulsions stabilized by prolate ellipsoids. It was found that both of them showed different aggregation morphology. One was preferentially aligned side by side, the other was preferentially arranged tip to tip, which is due to the different wettability of solid particles' surfaces with different chemical compositions. At the same time, they also found that the interaction force of the ellipsoid at the oil-water interface is 10^5^ times higher than the internal energy, while the strong interaction force of the regular sphere was weak or non-existent. Further, the study found that the strong interaction force is anisotropic. Subsequently, Loudet et al. explored the wetting and contact lines of ellipsoid particles (50). It is apparent that the interface is pulled down near the tips of the ellipsoid and pulled up near the middle of the particle (Figure 3A). The contact lines were saddle-shaped, and the deformation increased with the increase of the aspect ratio, while no such phenomenon was found in the contact lines of regular spheres. The deformation of the air-water interface was reported in many pieces of research. The shape of the contact line is determined by the contact angle, but the contact angle of the ellipsoid is not fixed. The contact angle at the tip of ellipsoid particles is smaller than that in the middle. Liu et al. (47, 75) first stabilized emulsion with food-grade cellulose nanocrystals (CNCs), shaped like short needles (about 200 nm long and 10 nm wide), and modified the CNCs with different concentrations of bovine serum protein (BSA about 500 nm long and 30 nm wide). This kind of particle can decrease the strong hydrophilicity of pure CNCs effectively. It was found that HIPEs could be stabilized by CNCs which are covered with BSA. It can form a bridge, and the hardness and microstructure of HIPEs could be adjusted by changing the concentration of CNCs.

### Nanofibrils

In addition to the above mentioned rod-like particles, nanocellulose can also be made into nanofibrils particles (Figure 3B). The most significant difference between nanofibrils and rod-like particles is that the former solid particles have a much larger aspect ratio, forming strong entangled and disordered network structures (83), which provides suitable conditions for stabilizing Pickering emulsion. Winuprasith et al. (52) extracted MFC from mangosteen rind to stabilize O/W Pickering emulsions and found that all emulsions remained stable at any concentration of MFC in the experiment, for the fact that at the oil-water interface the MFC may occur as single, dispersed fibers as well as in networks (84). Souza et al. (85) stabilized Pickering emulsions to prevent different essential oils (cinnamon, cardamom, and sapwood) volatilization with cellulose nanofiber (CNF) because CNF had the greatest coverage, the strongest network structure around cardamom oil droplets. In summary, nanofibrils particles tend to form three-dimensional networks structures on the surface of oil droplets and improve the mechanical properties of the emulsion. Besides, an elastic monolayer structure will be produced (86).

### Nanocages

At present, cage-like food-grade particles are mostly new-type nanocages with hollow interiors and porous wall surfaces prepared with protein. Nanocages are similar to spheres so the stability mechanism of nanocages resembles that of spherical particles (22). Sarker et al. (54, 87) prepared dodecahedral hollow protein nanocages using E2 protein to stabilize pH-responsive Pickering emulsions and proved that the cage-shaped solid particles show assembled structure of approximately 25 nm in diameter with 12 openings of about 5 nm each (Figure 3C).

### Plate-Shaped and Nanotubes

Due to the complicated structure of the protein, it is difficult to prepare disk solid particles (Figure 3D). Hikima et al. (55, 56) prepared Pickering emulsions with disk-like wool keratin nanoparticles by alkaline hydrolysis. α-lac-protein (α-lac) nanotubes (NTs, Figure 3E) formed by self-assembly of partially hydrolyzed α-lac-protein (α-lac) peptides were applied as an immobilization carrier for lipases (57), and to stabilize O/W Pickering emulsion by lipase-nanotube, which improved lipase activity, promoted milk fat hydrolysis and increased fatty acid release. In this way, it enhanced the flavor of the cheese.

### Other Irregular Shapes

In addition to the common shapes mentioned above, particles can also be shaped as spore particles (59), scaly (60), and so on. Binks et al. (59) investigated the potential of spore particles with rough surface structure and the presence of a Y-shaped marking (Figure 3F) of Lycopodium clavatum solely stabilizing Pickering emulsion with oils varying polarity, which can form clusters and chains at the oil-water interface. Liu et al. (60) prepared phytosterol colloidal particles showing (Figure 3G) gel-like would provide a novel candidate for stabilization and delivery system for active substances.

## External Factors Affecting Pickering Emulsion Stability

The effectiveness of particle emulsifiers' ability is related to their wettability, so it is strongly influenced by the hydrophobicity of particles, therefore, it can influence the type of Pickering emulsion. In most cases, changing the surface chemistry with small molecules or polymers through chemical anchoring and physical adsorption can alter the wettability of particles. Nevertheless, the interaction between particles and amphiphiles renders particles' wettability tunable in *in situ* modification. Xiao et al. (88) summarized tailoring the wettability by surface chemistry and surface roughness for Pickering emulsions, emphasizing the effects of surface roughness. In addition to the wettability of particles, external factors also have a critical influence on the stability of emulsions, such as the pH of the emulsion, particle concentration, ionic strength, the corresponding particle size, droplet size of emulsion, the type and fraction of oil phase.

### pH

**Figure 4:**
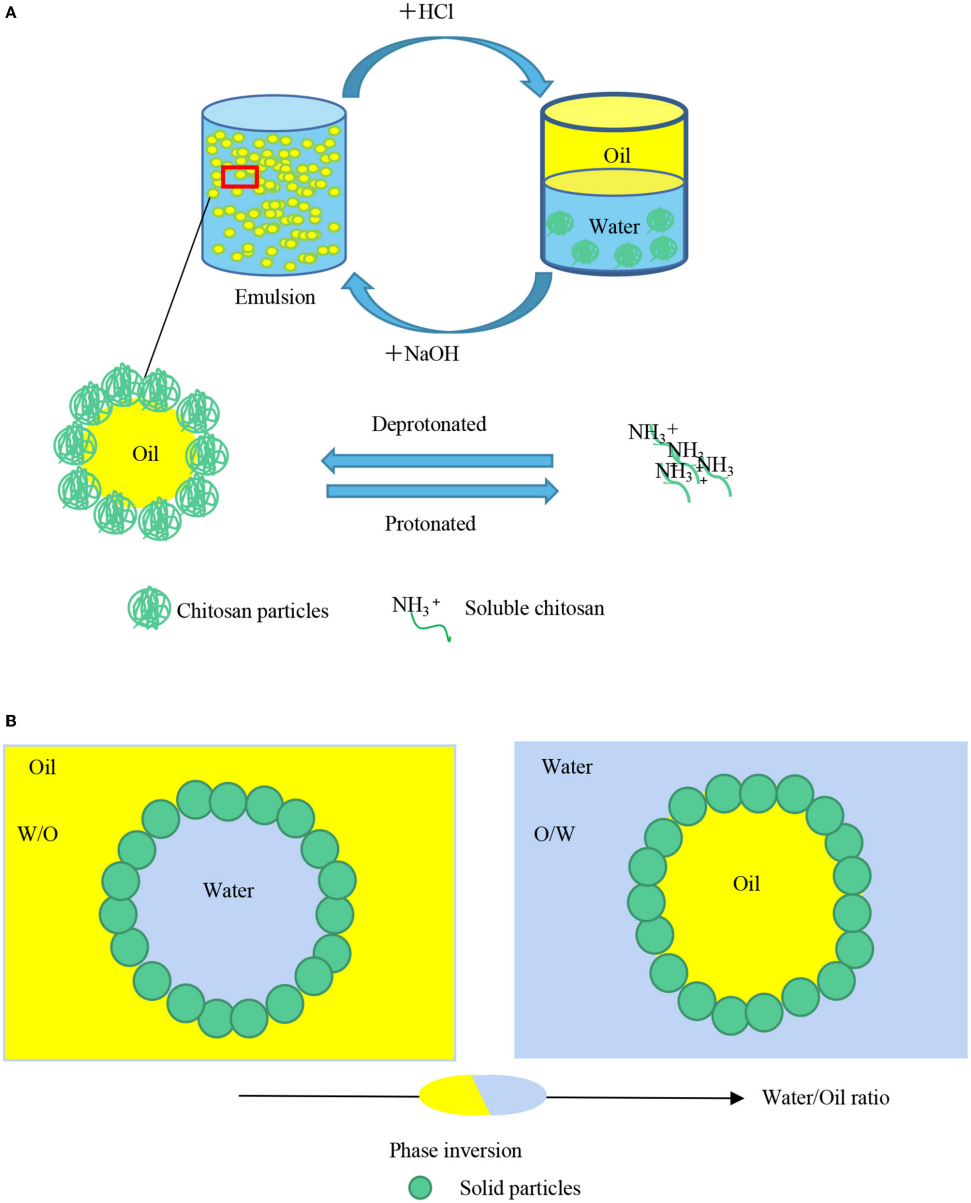
**(A)** External factors affect the stability of Pickering emulsions pH affects the electrostatic interaction among solid particles, causing to regulate the adsorption behavior of solid particles. The emulsion droplets will exhibit dispersion or flocculation with the different adsorption behaviors. **(B)** Phase inversion will happen when the oil radio increase from 0.2 to 0.6.

The pH of the system alters the surface charge of particles, hence affecting the electrostatic interaction among them, regulating the adsorption behavior as well as the space barrier formed at the oil-water interface. Therefore, the emulsion droplets exhibit dispersion or flocculation with the different adsorption behaviors and space barriers of particles (89, 90). Xiao et al. (91) stabilized Pickering emulsions with grafted carboxymethyl maize starch (CMS) nanoparticles showing pH-responsive properties. After imparted with amphipathic 2-(dimethylamine) ethyl methacrylate (DMAEMA) CMS exhibited different hydrophobicity at different pH values. Under acidic conditions, the protonated tertiary amine groups could decrease leading to a significant decrease in electrostatic interactions, hydrogen bonding, covalent bonding, and van der Waals forces. This resulted in particles separating from the two-phase interface into the oil phase. So the emulsions were unstable under acidic conditions. On the contrary, the nanoparticles can stabilize emulsions at pH 10. Li et al. (92), explored the influence of pH on the edible mayonnaise-like Pickering emulsions. They found the maximum droplet size did not appear at pI, but at pH 5.5, which could be due to the θ_O/W_ of 131.9 ± 0.6, which allowed the protein particles more easily to be wetted by the oil phase. Jiao et al. (93) found at pH 3.0, the droplet size is larger (5–50 μm,) than that at pH 9.0 (1–20 μm). Besides, at pH 9.0, the diameter of the peanut protein isolate microgel particles was around 270 nm and a larger interfacial area can be coated by them causing a smaller droplet size. Chitosan is the only natural positively charged polysaccharide, which can crosslink with the biomacromolecules such as proteins to prepare protein-chitosan particles due to its unique electrical properties free amino and hydroxyl groups carried along the backbone of the composite will be protonated or not by adjusting pH value of the system. Therefore, its adsorption quantity at the oil-water interface can be changed to realize the reversible process of emulsification-demulsification easily. Liu et al. (94) applied chitosan interaction to prepare pH-responsive Pickering emulsion. At pH > 6, chitosan nanoparticles or micrometer-sized floccular precipitates were formed and adsorbed on the interface to stabilize the emulsion. While at pH < 6, chitosan dissolved in water, and demulsification occurred (Figure 4A). Li et al. (95) prepared gliadin-chitosan nanoparticles (GCNPs) with three different structures, including primary complexation, soluble complexes, and coacervates by facile pH alteration. At pH 7.0, the coacervates type was prepared, owing to the decrease of the charge between the complexes and the weakening of the repulsive force. Droplet coalescence could be better prevented by the force on account of the highest viscoelastic and solid-like properties.

### The Concentration of Food-Grade Particles

The concentration of solid particles can influence the stability of the Pickering emulsion by affecting the droplet size of the emulsions, which is similar to the effect of surfactants on the stability of the traditional emulsions (96). It is generally considered that the final drop size of the Pickering emulsion virtually has nothing to do with the concentration of solid particles in excess (particles-rich regime at a high level) (97), which depended on the stirring intensity. However, if the concentration of solid particles keeps not very high (particles-poor regime, at a low level) the newly formed emulsion droplets only can be partly covered by solid particles. A number of droplets progressively combined with each other leading to the form of fast coalescence and large droplets. As a result, the final drop size is related to the concentration of solid particles. In contrast, with the increase in the concentration, more particles can adsorb at the interface to form a single or multi-layer structure, which prevents the coalescence of emulsion droplets (98, 99), thus stabilizing the emulsion. This is consistent with the finding of Wachira et al. (100). They proved that smaller oil droplet size could be caused by higher chitosan particle concentration, which increased larger surface area that reduced the collisions as well as movement between droplets. Concentration of particles can not only affect droplet size but also affect the structure of porous materials used to stabilize Pickering emulsions. Porous materials could be fabricated by the Pickering emulsion template method (93) whose thickness was able to tailor with various concentrations of the particles. At low particle concentration, the wall of the porous materials was fragile and shrunk. With increasing the concentration of the particles, the pore structure would be relatively uniform, and more stable emulsions could be obtained.

### Ionic Strength

Salt in the aqueous phase of the emulsion will produce an electrostatic shielding effect on the particles, leading to a change of particle surface charges and adsorption patterns at the interface, thus, changing the stability of the emulsion. Xiao (101), De Folter (102), and Liu (103) used kafirin nanoparticles, water-insoluble protein zein colloidal particles, and soybean protein to stabilize O/W emulsions, respectively. They found that within a certain range, with the increase of ionic strength of the system, the average droplet size decreased, and the emulsions tended to be more stable. The phenomenon is due to the shielding effect of ionic concentration within a certain range, which can enhance the interaction among particles, leading to the aggregation and migration of particles to the oil-water interface. Thus, the stability of the emulsion was improved. Addition to the alteration of the adsorption behaviors of particles at the oil-water interface, the ionic strength can also alter the hydrophobicity of particles. The ζ-potential of pea protein isolate microgel particle would decrease by increasing the concentration of NaCl, which may trigger the hydrophobic group to be embedded into protein curl, resulting in lower hydrophobicity (92).

### The Size of Solid Particles and Emulsion Droplets

The particle size of solid particles also influences the stability of the emulsions. Theoretically, a harder space obstacle can be formed with a decrease in particle size. So, it can make the solid particles adsorbed pretty closely at the water-oil interface (104), preventing the coalescence among droplets and the instability of emulsion. However, some studies have shown that particle size kept too large or too small may have an adverse effect on the stability of the emulsion. Ge et al. (105) found that the wettability of particles may be changed with the change in particle size. The Pickering emulsions stabilized by sweet potato and corn starch with a diameter ranging from 100–220 nm had much better stability than the particles with a diameter either less than 100 nm or larger than 220 nm. Additionally, the decrease in the size of droplets caused by increasing gel particles strength leads to maintain the stability of emulsions.

### Oil Type and Volume Fraction

The type of oil used for the preparation of Pickering emulsion and the radio of the dispersed and continuous phase play important roles in the stability of the emulsion. The oil type determines the interfacial tension of the droplets and the interactions among the particles on the two-phase interface can also be influenced. Tsuji et al. (106) reported that poly (N-isopropylacrylamide) (PNIPAM)-carrying particles tend to form O/W Pickering emulsions with varieties of oil types such as hexadecane, heptane, and trichloroethylene. This kind of oil phase all possesses the work of adhesion (Wa) ranging from 43 to 65 mJ/m^2^ related to the surface tension of water, O/W Pickering emulsions are preferably formed. The principle can be extended to food-grade emulsions. Zhang et al. (107) stabilized O/W Pickering emulsions, hexadecane/H_2_O, and decane/H_2_O Pickering emulsions, with poly (sodiump-styrenesulfonate) bush (PS@PSS), by ultrasonic power. The microscope images showed that droplets were significantly bigger and more homogeneous in hexadecane than in decane. When the oil fraction increased from 0.2 to 0.6, the emulsions type changed from O/W to W/O which was stabilized by the water-insoluble phytosterols (60) (Figure 4B).

### Temperature

The emulsifying temperature also has effects on food-grade Pickering emulsions. The nanoparticles had poor thermal stability, however, the thermal stability of Pickering emulsions improved with the application of the triple emulsifier (108). In the actual preparation process, the final stability of the emulsion is the result of the interaction of various factors.

## Applications In the Food Field

There have been some stable food-grade emulsions and they can be a potential candidate for promising functions. In the past few years, Pickering emulsions for their superior performance have been reported in cosmetics, pharmacy, tissue engineering, and the food field (15, 109–111). Especially, the edible particles are able to meet the demand for a “clean label”, These particles can act as either the direct stabilizer of Pickering emulsions or indirect materials fabricated by them.

### Pickering Emulsion for Functional Active Substances Encapsulation

**Figure 5:**
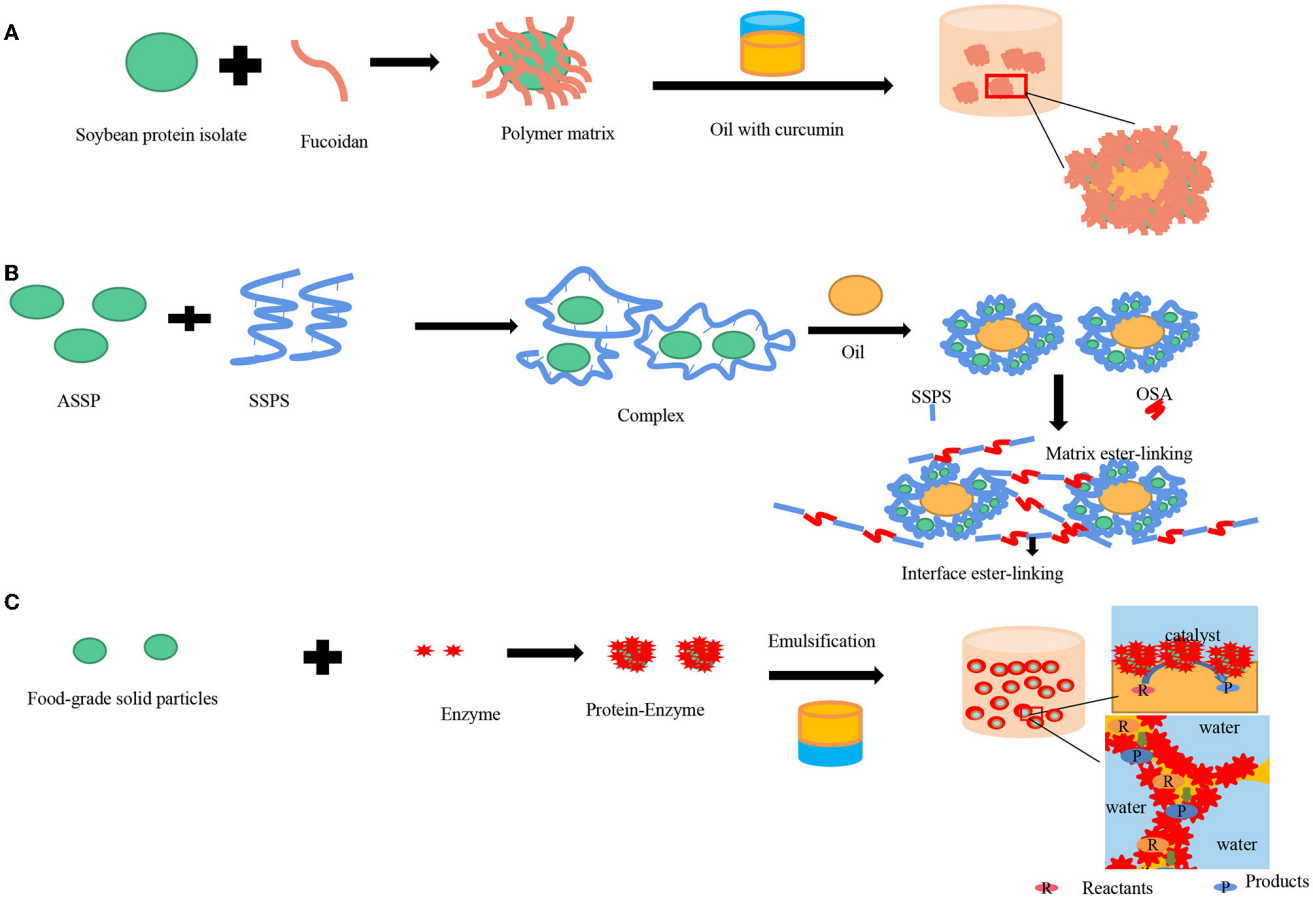
Pickering emulsions applied in foods fields. **(A)** Encapsulation of curcumin; **(B)** Food packaging film preparation; **(C)** Pickering emulsion catalytic efficiency improving.

Pickering emulsion is a good encapsulation agent, which can improve the storage stability of functional active substances and improve their bioavailability. Currently, it has been widely used to carry functional active substances in various fields (112). Based on Pickering emulsion, microcapsules and microspheres are commonly prepared to explore the utilities of a delivery system for bioactive compounds. By emulsifying, active ingredients can be firstly encased in the droplets. The interfaces of the droplets are further stabilized through interfacial assembly, deposition, and the shelled microcapsules can be fabricated by interfacial polymerization (113). According to the hydrophilicity or hydrophobic of functional activities, they can be encapsulated and delivered by Pickering emulsion of different types (O/W or W/O). Curcumin is a hydrophobic active substance extracted from turmeric rhizomes (114), which has a series of pharmacological activities such as anti-inflammatory, antibacterial, and antiulcer activities (115–117). However, the use of curcumin has been limited by low physicochemical stability and water solubility (118). Therefore, a wide variety of encapsulation delivery systems should be applied (118, 119) to improve its stability and solubility (Figure 5A). Shah et al. (120) prepared chitosan-tripolyphosphate nanoparticles (CS-TPP) by ionic gelation techniques to stabilize O/W Pickering emulsion, encapsulated curcumin, and evaluated the stability and release kinetics of curcumin based on spectrophotometry. The results showed that only 14% of curcumin was degraded within 24 h, which was significantly optimized compared with Leung et al. (121) used sodium dodecyl sulfate (SDS) encapsulation of curcumin, which degraded 40% in 20 h. In addition, the emulsions can also improve the stability of curcumin in acid, alkali, and salt existed environment. In addition to preparing nano-particle stabilized Pickering emulsions by ionic gelation techniques, Lv et al. (122) prepared whey protein isolate (WPI) gel by hydrostatic high-pressure treatment (HHP) for loading and wrapping curcumin. Since WPI could form a compact solid-like gel at pH 5, it has been verified by simulated gastrointestinal digestion experiments *in vitro* that the release rate of curcumin at pH 5 was slower than that at pH 6.0. On the other hand, because the non-thermal means were used to prepare gelation, it can effectively avoid the generation of undesirable odor. Li et al. (95) prepared Pickering emulsions stabilized by wheat gliadin-chitosan nanoparticles with three structures by changing pH and confirmed that the complex formed by wheat gliadin-chitosan and curcumin could effectively reduce lipid oxidation through accelerating thermal storage experiments. Hydrophilic activities such as lutein and anthocyanins can be delivered by W/O Pickering emulsion encapsulated in the water phase. Li et al. (123) stabilized Pickering emulsion gels by octenylsuccinate quinoa starch (OSQS) with different interface activities and structures by adjusting the oil phase volume fraction. This emulsion was used to deliver, and the retention rate of lutein could keep 55.38% after 31 days of storage. Furthermore, multiple Pickering emulsions due to their function of isolation, protection, and targeted release have been used to encapsulate active ingredients to improve their stability to light and oxygen (124). Beicht et al. (125) fabricated multiple emulsions by fish gelatin (FG), whey protein isolate (WPI), and dodecyltrimethylammonium bromide (DTAB), which can also improve the stability of lutein obviously. Wang et al. (126) formulated water-in-oil-in-water (W/O/W) emulsions to encapsulate trans-resveratrol in the internal water phase and oil phase together with an encapsulation efficiency of 99.97 ± 0.001%.

### Pickering Emulsion for Existing Food System Replacing

In recent years, the consumption of margarine, shortening, and so on showed a steady growth trend with the pursuit of diverse food and the demand for various special fats. However, most of the edible oil has to be commercially produced by the hydrogenation process, which produced a large number of trans-fatty acids (TFA). It is reported (127, 128) that TFA can increase the incidence of myocardial infarction and coronary heart disease. Therefore, it is crucial to find substitutes for partially hydrogenated vegetable oils. Jiao (93) et al. stabilized a high internal phase Pickering emulsion solely by peanut protein microgel particle whose high viscosity is similar to that of cream, which is expected to become the substitution for margarine containing partially hydrogenated oils. Li et al. (92) fabricated edible egg-free mayonnaise-like Pickering emulsion by pea protein isolate microgels, which showed similar properties to commercial mayonnaise. Especially, the thixotropy recovery rate can achieve nearly 100%. Typical effects on ζ-potential, the droplet size of the emulsion, such as pH, NaCl, and sucrose, were explored. This is the first study to scale up the processing of mayonnaise-like Pickering emulsion manufacturing at a pilot-plant scale. Besides many researchers (91, 129–132) also have made mayonnaise-like Pickering emulsion based on yolk-free materials. Romoscanu et al. (133) prepared a kind of low-viscosity liquid and oleogels based on the protein-template method. It avoided the hydrogenation process and thus TFA would not be produced. Besides, the oleogels had a viscoelastic and crystal structure similar to that of solid fat, which would play a great potential role in the food industry. The study found that (134) nanocellulose can change the rheological properties of liquid food and cellulose nanofiber (CNFs) because of its high transverse and longitudinal. Under the condition of low concentration, nanofibrils can form a gel (135). Meanwhile, the quality and structure strength of the gel can be changed (136). The gel strength will increase, and the gel will keep transparent with surfactants added to it. Conversely, the gel strength will decrease when the surfactant concentration gets high. Velásquez-Cock et al. (137) substituted fat with CNFs to improve the melting physical characteristics of ordinary fat ice cream and found that the melting rate of ordinary ice cream could be reduced with the increase of CNFs content. When the CNFs content reached the maximum, the melting rate decreased by 50%. However, the melting rate of the low-fat ice cream was significantly reduced only when the CNFs content reached 0.3%. This is related to water retention and lipid retention of hydroxyl and amino groups in the mixed system (138). Wang et al. (139) reported a facial method where Pickering emulsion-template was used to stabilize the Pickering emulsion *via* zein/chitosan colloidal particles (ZCCP) which can transform the liquid oil into solid fat, potential antibacterial and antioxidant properties.

### Pickering Emulsion for Food Packaging Film Preparation

Plastic products have brought great convenience to people's daily life and production-manufacturing since their appearance, but they also produced inevitable negative effects. So far, only 9% of plastic in the world can be recycled, and most of the plastic in the environment is not biodegradable, posing a great threat to animals, plants, and humans. Recently, the use of edible films prepared by using polysaccharides, proteins, and lipids instead of plastic products has attracted considerable attention. Compared with traditional edible films, emulsion films combine the advantages of hydrocolloid (polysaccharide or protein) and lipophilic lipids, which can be used as an effective control barrier for flavor and moisture (140). Among them, Pickering films are synthesized by adding emulsifiers to the lipid in combination with polysaccharides or proteins forming homogeneous emulsions to form Pickering films (141). Konjac glucomannan (KGM) has been considered as a promising material to fabricate Pickering films for its biocompatibility as well non-toxicity (142). Liu et al. (143) investigated the emulsion particle size that played an important role in the properties of KGM-based films. The properties of KGM-based films were improved by incorporating Pickering emulsions with various particle sizes. Some researchers prepared Pickering film encapsulation volatiles by combining polylactic acid film with nanocellulose (144) forming a complementary film. Soluble soybean protein-polysaccharide (SSPS) linked with acid-soluble soybean protein (ASSP) (145) was also used to prepare food film (Figure 5B). The membrane has good barrier performance of moisture and oxygen. Furthermore, it has been proved that incorporating essential oil, bioactive compounds, and other functional composition into polysaccharide- or protein-based films could be a promising method to improve the antimicrobial and antioxidant abilities of the films (146).

### Pickering Emulsion for Catalytic Efficiency Improving

The conventional biphasic system (BS) exhibits poor catalysis efficiency, due to the low contact area between the immiscible phases, thus limiting the reaction and production. Pickering emulsions as the carrier of catalytic reaction can be a promising strategy to preferably improve the efficiency of the catalysis reaction, which not only has high catalytic efficiency and selectivity but also is conducive to the recovery and utilization of solid particles and catalysts. It is generally recognized that the interface is significant for enzyme activity because the reaction often occurs at the oil-water interface in Pickering emulsion catalytic systems. Generally, for Pickering emulsion catalysis, it is not necessary to add external force for mechanical mixing, and no traditional surfactant is used in classical emulsions, thus adding particles to divide one phase into droplets within micrometers in another immiscible phase. In this way, as the numerous droplets act as micro-compartments, a dramatically large interface area can be provided, thus enhancing the encounter of the enzyme and substrate. At the same time, because of the existence of the microdroplets, the transfer distance between the catalyst and reactant can be shortened (Figure 5C). Nardello-Rataj et al. (147) defined the Pickering emulsion catalysis (PEC) as two types, Pickering Assisted Catalysis (PAC) as well as Pickering Interfacial Catalysis (PIC). For PAC, the catalysts can be confined in the organic or aqueous phase not be used to stabilize Pickering emulsions. For PIC, Pickering emulsions will be stabilized by catalysts combined with solid particles. In recent years, PEC has been applied in the food field although the particles are not food-grade (118, 148–153). At present, there exist Pickering emulsions stabilized by edible particles used in the food field to improve the catalytic efficiency of lipases, such as hydrolysis, esterification, and deacidification. The wettability of chitosan was changed *in situ* modification, which can lead to the interaction triggered by lipase between the free fatty acid and the nanogel particles (118). In this experiment, genipin was used as a cross-linking reagent for lacking physiological toxicity. After 13 batches, the activity of the Pickering emulsion can still remain at 55% of the initial system. For generating free fatty acids, the emulsion would undergo an inversion of phase thus facilitating the separation of products and reactants. Another study (57) used partially hydrolyzed α -whey protein (α-LAC) peptides to form α-whey protein nanotubes (NTs) and immobilized lipase to improve the catalytic activity of lipase. It was found that free fatty acids hydrolyzed by lipase-NTS were 1.5 times as much as those hydrolyzed by free lipase.

## Conclusion and Outlook

Food-grade particles stabilized Pickering emulsion has the advantages of environmental friendly, biocompatibility, stable property. Based on their natural properties and feasible modification, they have been widely used in food science. Either stabilizing mechanism or food application to Pickering emulsions stabilized by spherical particles has been mature and widely applied. However, there still exist some challenges in implementing edible Pickering emulsions in commercial applications. In addition, it is expected that understanding materials and applications from the state-of-art technology will enable researchers to modify and design more innovative formulations, which could be more beneficial for exploring food production and processes. Several types of studies should be further explored in the future. (1) Compared with spherical food-grade particles, the mechanism and application of Pickering emulsions stabilized by non-spherical particles need further exploration. The researchers are still required to identify how the nature, application, and stability are related to the shape of particles. More accurate approaches should be adopted to study the interfacial behavior of these irregular shape particles. Without particles adsorbing at the interface of two phases, the interfacial layers affected by the order of addition are worth investigating which can cause diverse interfacial structures (154). (2) Due to the high sensitivity and low bioavailability of bioactive compounds, Pickering emulsions stabilized by hybrid biopolymeric particles have attracted researchers' attention. But there is still a lack of detailed research on the toxicity and allergy of biopolymers although they exhibit a lot of advantages in the food field (155). Besides, the encapsulation efficiency and loading capacity of the Pickering emulsions should be considered. Thus, it is also important to carry out *in vivo* studies to validate their biological activities. (3) To attain excellent sensorial pleasure, the oral tribology and sensory perception of the Pickering emulsion will also increasingly deserve attention. Generally speaking, for consumers, the rheological and tribological properties of emulsion play a prime role in sensory perception during oral processing (131). So, it is urgent to improve the rheological and tribological properties to improve the palatability of food-based Pickering emulsion. (4) Although we have a relatively clear understanding of the mechanism of HIPEs, the applications of HIPEs still need to be explored, such as desired porous materials. The porous materials can be applied to medical and tissue engineering for moderate and natural crosslinking ways. Besides, the porous materials are to expected modify and design functional materials with exquisite structure and responsiveness. The construction of the porous scaffolds is expected to control the release of drugs and bioactives. (5) There are urgent needs for commercially viable plant-scale manufacturing techniques and commercially available food products based on Pickering emulsions. In practice, matching techniques in lab-scale to real plant-scale processes or equipment is very challenging. (6) More edible particles rather than inorganic solid particles should be explored and applied to form Pickering emulsion catalysis system to achieve high enzyme activity and recovery efficiency in food industry. These edible particles will provide more sustainable and safer for food products.

## Author Contributions

WL and BJ: conceptualization and writing—original draft. SL: data curation. SF: grammar. WF and YC: polish. AS and QW: supervision. QW: visualization. All authors read and approved the submitted version of the manuscript.

## Funding

This research was funded by the National Natural Science Foundation of China (U21A20270 and 32172149), China Postdoctoral Science Foundation (2020M680777 and 2021T140717), and the Agricultural Science and Technology Innovation Program of the Chinese Academy of Agricultural Sciences (CAAS-ASTIP-2020-IFST).

## Conflict of Interest

The authors declare that the research was conducted in the absence of any commercial or financial relationships that could be construed as a potential conflict of interest.

## Publisher's Note

All claims expressed in this article are solely those of the authors and do not necessarily represent those of their affiliated organizations, or those of the publisher, the editors and the reviewers. Any product that may be evaluated in this article, or claim that may be made by its manufacturer, is not guaranteed or endorsed by the publisher.
